# Clinicopathological characteristics and outcomes in stage I–III mucinous gastric adenocarcinoma: a retrospective study at a single medical center

**DOI:** 10.1186/s12957-016-0886-5

**Published:** 2016-04-26

**Authors:** Jun-Te Hsu, Chen-Wei Wang, Puo-Hsien Le, Ren-Chin Wu, Tsung-Hsing Chen, Kun-Chun Chiang, Chun-Jung Lin, Ta-Sen Yeh

**Affiliations:** Department of General Surgery, Chang Gung Memorial Hospital at Linkou, Chang Gung University College of Medicine, 5, Fushing Street, Kweishan District, Taoyuan City, 333 Taiwan; Department of Gastroenterology, Chang Gung Memorial Hospital at Linkou, Chang Gung University College of Medicine, Taoyuan, Taiwan; Department of Pathology, Chang Gung Memorial Hospital at Linkou, Chang Gung University College of Medicine, Taoyuan, Taiwan; Department of Surgery, Chang Gung Memorial Hospital at Keelung, Chang Gung University College of Medicine, Taoyuan, Taiwan

**Keywords:** Mucinous gastric cancer, Undifferentiated, Prognostic factor

## Abstract

**Background:**

The clinicopathological characteristics and outcomes of mucinous gastric adenocarcinoma (GC) remain unclear. We report the clinicopathological features and prognosis of patients with mucinous histology who underwent radical-intent gastrectomy.

**Methods:**

We reviewed the medical records of 1470 patients with pathologically proven undifferentiated GC undergoing radical-intent gastrectomy between 1995 and 2007. The patients were stratified into three groups according to their histological type: mucinous carcinoma (MC), signet ring cell carcinoma (SRCC), and poorly differentiated carcinoma (PDC). Clinicopathological factors affecting prognosis were collected prospectively and analyzed.

**Results:**

In stage III MC, the age and size were significantly greater and larger than in SRCC and PDC; a lower proportion of perineural invasion was identified in MC, and female predominance was noted in SRCC in comparison with MC and PDC. The cumulative overall survival rates of stage I–III GC patients with MC were significantly superior compared to those with PDC, but not SRCC. Stage III GC patients with MC had a better prognosis than those with SRCC or PDC; the difference in survival was not evident in stages I or II.

**Conclusions:**

Thus, MC presents with different clinicopathological features and prognosis from SRCC and PDC. The patients with stage III gastric MC had favorable outcomes.

## Background

Despite the decreasing overall worldwide incidence of gastric adenocarcinoma (GC), GC has remained the third leading cause of cancer-related death, after lung and liver malignancies, leading to around 723,000 deaths in 2012 [1]. Most GC patients present with locally advanced or metastatic disease, and radical surgical resection is still the mainstay of treatment for localized disease [2, 3]. According to the Japanese classification for histological typing for GC, mucinous carcinoma (MC) or signet ring cell carcinoma (SRCC) is defined as the undifferentiated type [4]. Undifferentiated carcinoma also has different biological behaviors than differentiated carcinoma, such as the growth pattern, invasiveness, metastasis, and prognosis [5]. However, even between tumors belonging to the undifferentiated histology subtype, there can be significant heterogeneity in terms of tumor biology and prognosis. Studies reported that MC accounts for 2.6–7.6 % of all GC [6]. Only a few studies on gastric MC have been reported, and its clinicopathological features and prognosis were inconsistent [5–7]. For example, Yin et al. indicated that there was no difference in survival between MC and non-MC [6]. However, Kunisaki et al. observed that MC had a poor prognosis compared with non-MC [7]. The aims of this study were to elucidate the clinicopathological characteristics and to clarify the prognosis of stage I–III resected GC patients with MC compared with other undifferentiated subtypes.

## Methods

### Ethics statement

The study protocol was approved by the Institutional Review Board of Chang Gung Memorial Hospital (No. 100-4279B). Written informed consent was obtained from all the patients. All data were stored in the hospital database and used for research.

### Patients and surgical procedures

We reviewed the medical records of 1470 patients with pathologically proven undifferentiated GC undergoing radical-intent gastrectomy at Chang Gung Memorial Hospital, Taoyuan, Taiwan, between 1995 and 2007; patients with a history of partial gastrectomies were excluded. The patients were stratified into three groups according to the histological types: MC, SRCC, and poorly differentiated carcinoma (PDC). Subtotal or total gastrectomy was performed according to the tumor size, location of tumor, and status of resection margins. The standard procedure included a spleen- and pancreas-preserving D1 or D2 lymph node dissection, depending on the perceived extent of tumor invasion and lymph node metastasis [2]. Resection of adjacent organs was undertaken to achieve clear margins when deemed necessary [8]. Surgery-related complications included anastomotic/duodenal stump leakage, wound infection, intra-abdominal abscess/bleeding, and delayed gastric emptying, while pneumonia, cardiovascular event, atelectasis, sepsis, paralytic ileus, pleural effusion, urinary retention, and psychoneurologic event were considered as surgery-unrelated complications. Lymphatic, vascular, or perineural invasion was defined as the presence of permeation of the tumor in the lymphatic duct, vascular structure, or nerve microscopically, respectively. The tumors were staged according to the seventh edition of the American Joint Committee on Cancer Tumor Node Metastasis classification [9]. Postoperative adjuvant chemotherapy with fluoropyrimidine-based or platinum-based regimens was indicated for patients with stage II–III disease, while patients with stage IB did not routinely received adjuvant chemotherapy except for those with tumors showing poor differentiation or lymphatic, vascular, or perineural invasion. No patient received neoadjuvant chemotherapy. The median follow-up time was 41.0 months, ranging from 1.2 to 215.9 months. The patients who died after surgery during the same hospitalization were defined as hospital mortality and were not included in the long-term survival analysis. Survival duration was calculated from the time of surgery to death or the last follow-up date (December 31, 2012), irrespective of the cause of death.

### Statistical analysis

Clinical records were compared with either Fischer’s exact test or Pearson’s *χ*^2^ test, as appropriate. The patient survival rate was calculated using the Kaplan-Meier curve, and univariate analysis was conducted using the log-rank test. Factors that were deemed of potential importance to the univariate analysis (*P* < 0.05) were included in the multivariate analysis using the Cox regression model. *P* < 0.05 was considered significant. Statistical analyses were performed with SPSS software for Windows, version 13 (SPSS, Inc., Chicago, IL, USA).

## Results

### Demographics and clinicopathological data

Table 1 shows the demographics and clinicopathological features of patients with stage I–III undifferentiated GC who underwent potentially curative gastrectomy stratified according to histology type. No difference was noted in terms of the extent of lymphadenectomy, number of lymph nodes retrieved, resection margins, surgery-related complications, and hospital mortality among the three groups. Older patients (*P* < 0.0001), larger tumor size (*P* < 0.0001), higher incidences of stage III tumors (*P* < 0.001), higher total complication rates (*P* = 0.016), and higher percentages of patients receiving adjuvant chemotherapy (*P* = 0.047) were observed in the group of patients with MC histology than in those with the SRCC or PDC subtypes. Compared with MC and PDC, SRCC had female predominance (*P* < 0.001); higher incidences of T4 tumors (*P* < 0.0001); and greater lymphatic (*P* < 0.0001), vascular (*P* = 0.005), or perineural (*P* = 0.001) invasion. More total gastrectomy procedures (*P* = 0.006), cases with N3 status (*P* < 0.0001), and surgery-unrelated complications (*P* = 0.031) were noted in the PDC subtype than in SRCC and MC. In stage III undifferentiated GC patients, MC had older patients (*P* = 0.019), larger tumor size (*P* = 0.006), and more perineural (*P* = 0.003) invasion than SCC and PDC (Table 2).

**Table 1:** Clinicopathological characteristics of stage I–III undifferentiated gastric adenocarcinoma in terms of histology type

Parameters	MC (*n* = 54)	SRCC (*n* = 545)	PDC (*n* = 871)	*P* value
Age (years), mean ± SD	64.2 ± 10.7	58.4 ± 13.4	62.5 ± 13.8	<0.0001
Median	66.0	59.0	65.0	
Sex				<0.001
Male	31 (57.4)	269 (49.4)	522 (59.9)	
Female	23 (42.6)	276 (50.6)	349 (40.1)	
Tumor size (cm), mean ± SD	6.4 ± 3.9	4.3 ± 3.4	5.1 ± 3.2	<0.0001
Median	5.5	3.5	4.5	
Type of gastrectomy				0.006
Total	16 (29.6)	133 (24.4)	282 (32.4)	
Subtotal	38 (70.4)	412 (75.6)	589 (67.6)	
Extent of LN dissection				0.773
<D2	21 (38.9)	187 (34.3)	308 (35.4)	
≥D2	33 (61.1)	358 (65.7)	563 (64.6)	
No. of LN retrieval, mean ± SD	27.3 ± 15.8	28.1 ± 15.3	27.7 ± 14.7	0.856
Median	23.0	25.0	25.0	
T status				<0.0001
T1	2 (3.7)	163 (29.9)	101 (11.6)	
T2	9 (16.7)	56 (10.3)	74 (8.5)	
T3	3 (5.6)	19 (3.5)	24 (2.8)	
T4	40 (74.1)	307 (56.3)	672 (77.2)	
LN status				
N0	13 (24.1)	251 (46.1)	244 (28.0)	<0.0001
N1	11 (20.4)	59 (10.8)	119 (13.7)	
N2	12 (22.2)	66 (12.1)	56 (17.9)	
N3	18 (33.3)	169 (31.0)	352 (40.4)	
Stage				<0.0001
I	5 (9.3)	190 (34.9)	120 (13.8)	
II	10 (18.5)	94 (17.2)	170 (19.5)	
III	39 (72.2)	261 (47.9)	581 (66.7)	
Positive margins (R1 resection)	8 (14.8)	59 (10.8)	95 (10.9)	0.662
Lymphatic invasion^a^				<0.0001
No	21 (39.6)	288 (53.4)	315 (36.7)	
Yes	32 (60.4)	251 (46.6)	544 (63.3)	
Vascular invasion^a^				0.005
No	44 (83.0)	479 (89.2)	710 (82.9)	
Yes	9 (17.0)	58 (10.8)	146 (17.1)	
Perineural invasion^a^				0.001
No	27 (51.9)	294 (54.9)	380 (44.3)	
Yes	25 (48.1)	242 (45.1)	477 (55.7)	
Complications^b^	12 (22.2)	61 (11.2)	136 (15.6)	0.016
Surgery-related	10 (18.5)	49 (9.0)	99 (11.4)	0.064
Leakage	5 (9.3)	26 (4.8)	46 (5.3)	
Intra-abdominal abscess	4 (7.4)	18 (3.3)	38 (4.4)	
Wound infection	1 (1.9)	5 (0.9)	22 (2.5)	
Bleeding	1 (1.9)	9 (1.7)	11 (1.3)	
Delayed gastric emptying	0	1(0.2)	6 (0.7)	
Others	1 (1.9)	3 (0.6)	4 (0.5)	
Surgery unrelated	3 (5.6)	19 (3.5)	59 (6.8)	0.031
Pneumonia	1 (1.9)	2 (0.4)	15 (1.7)	
Cardiovascular event	1 (1.9)	5 (0.9)	7 (0.8)	
Sepsis	0	2 (0.4)	8 (0.9)	
Paralytic ileus	0	2 (0.4)	5 (0.6)	
Atelectasis	0	0	6 (0.7)	
Others	4 (7.4)	21 (3.9)	45 (5.2)	
Hospital mortality	4 (7.4)	14 (2.6)	33 (3.8)	0.130
Chemotherapy^c^	38 (84.4)	285 (81.7)	553 (75.6)	0.047

Figures are numbers with percentages in parentheses, unless otherwise stated

*LN* lymph node, *MC* mucinous carcinoma, *PDC* poorly differentiated carcinoma, *SD* standard deviation, *SRCC* signet ring cell carcinoma

^a^Some data were missing

^b^Number of patients with event

^c^Excluding T1/T2N0 cases or hospital mortality

**Table 2:** Clinicopathological characteristics of stage III undifferentiated gastric adenocarcinoma in terms of histology type

Parameters	MC (*n* = 39)	SRCC (*n* = 261)	PDC (*n* = 581)	*P* value
Age (years), mean ± SD	64.5 ± 10.0	60.2 ± 13.7	62.7 ± 14.0	0.019
Median	66.0	61.0	66.0	
Sex				0.002
Male	22 (56.4)	128 (49.0)	359 (61.8)	
Female	17 (43.6)	133 (51.0)	222 (38.2)	
Tumor size (cm), mean ± SD	7.6 ± 3.9	6.0 ± 3.7	5.8 ± 3.1	0.006
Median	7.0	5.0	5.0	
Type of gastrectomy				0.696
Total	13 (33.3)	96 (36.8)	226 (38.9)	
Subtotal	26 (66.7)	165 (63.2)	355 (61.1)	
Extent of LN dissection				0.268
<D2	13 (33.3)	71 (27.2)	190 (32.7)	
≥D2	26 (66.7)	190 (72.8)	391 (67.3)	
No. of LN retrieval, mean ± SD	29.2 ± 16.3	30.2 ± 16.2	28.6 ± 14.9	0.373
Median	25.0	27.0	26.0	
T status				0.627
T2	2 (5.1)	6 (2.3)	10 (1.7)	
T3	1 (2.6)	5 (1.9)	9 (1.5)	
T4	36 (92.3)	250 (95.8)	562 (96.8)	
LN status				
N0	2 (5.1)	1 (0.4)	13 (2.2)	0.118
N1	8 (20.5)	41 (15.7)	86 (14.8)	
N2	11 (28.2)	52 (19.9)	136 (23.4)	
N3	18 (46.2)	167 (64.0)	346 (59.6)	
Positive margins (R1 resection)	8 (20.5)	48 (18.4)	84 (14.5)	0.255
Lymphatic invasion^a^				0.442
No	8 (21.1)	35 (13.5)	88 (15.4)	
Yes	30 (78.9)	225 (86.5)	484 (84.6)	
Vascular invasion^a^				0.767
No	30 (78.9)	204 (79.1)	439 (76.9)	
Yes	8 (21.1)	54 (20.9)	132 (23.1)	
Perineural invasion^a^				0.003
No	17 (45.9)	57 (22.2)	175 (30.8)	
Yes	20 (54.1)	200 (77.8)	394 (69.2)	
Complications^b^	8 (20.5)	44 (16.9)	101 (17.4)	0.854
Surgery-related	8 (20.5)	35 (13.4)	71 (12.2)	0.316
Leakage	4 (10.3)	19 (7.3)	34 (5.9)	
Intra-abdominal abscess	4 (10.3)	12 (4.6)	28 (4.8)	
Wound infection	1 (2.6)	4 (1.5)	15 (2.6)	
Bleeding	1 (2.6)	8 (3.1)	9 (1.5)	
Delayed gastric emptying	0	1 (0.4)	4 (0.7)	
Others	0	3 (1.1)	3 (0.5)	
Surgery unrelated	1 (2.6)	16 (6.1)	48 (8.3)	0.275
Pneumonia	0	2 (0.8)	12 (2.1)	
Cardiovascular event	0	5 (1.9)	6 (1.0)	
Sepsis	0	2 (0.8)	7 (1.2)	
Paralytic ileus	0	1 (0.4)	4 (0.7)	
Atelectasis	0	0	5 (0.9)	
Others	1 (2.6)	18 (6.9)	38 (6.5)	
Hospital mortality	2 (5.1)	13 (5.0)	32 (5.5)	0.950
Chemotherapy	30 (76.9)	205 (78.5)	423 (72.8)	0.197

Figures are numbers with percentages in parentheses, unless otherwise stated

*LN* lymph node, *MC* mucinous carcinoma, *SD* standard deviation, *PDC* poorly differentiated carcinoma, *SRCC* signet ring cell carcinoma

^a^Some data were missing

^b^Number of patients with event

### Analysis of prognostic factors

Univariate analysis indicated that the type of gastrectomy; ratio of metastatic to examined lymph nodes; nodal status; histology type; resection margins; presence of lymphatic, vascular, and perineural invasion; and patients receiving adjuvant chemotherapy were significant prognostic factors for stage III undifferentiated GC patients (Table 3). Multivariate analysis showed that the following factors significantly affected survival in stage III undifferentiated GC patients: type of gastrectomy (total vs. subtotal; hazard ratio (HR) = 1.130; *P* = 0.001), tumor size (>5 cm vs. ≤ cm; HR = 1.251; *P* = 0.013), ratio of metastatic to examined lymph nodes (>0.34 cm vs. ≤0.34 cm; HR = 1.892; *P* < 0.0001), positive resection margins (HR = 1.238; *P* < 0.001), histology type (PDC vs. MC; HR = 1.594; *P* = 0.04), the presence of perineural invasion (HR = 1.335; *P* = 0.004), and no administration of chemotherapy (HR = 1.381; *P* = 0.002) (Table 4).

**Table 3:** Univariate analysis of prognostic factors for stage III undifferentiated gastric adenocarcinoma

Factors	Median survival (months)	95 % CI for median	3-year survival (%)	5-year survival (%)	*P* value
Age (years)					0.200
≤65 (*n* = 449)	23.8	20.4–27.3	39.2	29.2	
>65 (*n* = 385)	21.8	19.0–24.6	33.5	24.1	
Sex					0.374
Male (*n* = 474)	21.8	19.1–24.6	34.7	25.8	
Female (*n* = 360)	25.2	21.3–29.2	38.3	28.6	
Tumor size (cm)					0.223
≤5 (*n* = 410)	28.4	23.9–33.0	43.1	32.9	
>5 (*n* = 420)	19.0	16.6–21.4	29.2	21.6	
Type of gastrectomy					<0.0001
Total (*n* = 308)	17.8	15.0–20.7	29.6	20.6	
Subtotal (*n* = 526)	26.6	23.1–30.1	40.1	30.7	
Extent of LN dissection					0.834
<D2 (*n* = 246)	24.3	19.6–28.9	36.2	27.3	
≥D2 (*n* = 588)	22.2	19.3–25.2	36.2	26.9	
T status					0.817
T1/T2 (*n* = 17)	17.6	5.6–9.6	41.2	32.9	
T3/T4 (*n* = 817)	23.0	20.5–25.6	36.2	26.9	
LN ratio					<0.0001
≤0.34 (*n* = 416)	38.7	30.8–46.7	52.4	41.9	
>0.34 (*n* = 418)	15.8	13.5–18.1	20.1	12.0	
N status					<0.0001
N0 (*n* = 15)	NA		57.8	57.8	
N1 (*n* = 129)	65.3	29.5–101.1	58.9	50.3	
N2 (*n* = 188)	28.6	20.1–37.1	44.3	33.8	
N3 (*n* = 52)	18.8	16.7–0.9	26.5	17.3	
Histology type					0.038
MC (*n* = 37)	47.8	0.0–103.0	51.1	48.1	
SRCC (*n* = 248)	20.8	16.5–25.0	36.9	26.0	
PDC (*n* = 549)	22.3	19.5–25.1	34.9	26.1	
Resection margins					<0.0001
R0 (*n* = 709)	25.4	22.4–28.3	39.8	30.1	
R1 (*n* = 125)	15.8	12.7–18.9	15.3	8.6	
Lymphatic invasion					0.006
No (*n* = 125)	33.1	23.4–42.8	45.6	36.0	
Yes (*n* = 698)	21.5	19.5–23.6	34.1	25.0	
Vascular invasion					0.024
No (*n* = 635)	24.3	21.7–26.9	37.2	28.1	
Yes (*n* = 185)	18.5	14.7–22.2	31.7	23.2	
Perineural invasion					<0.0001
No (*n* = 234)	31.4	19.1–43.8	46.9	40.4	
Yes (*n* = 582)	20.8	18.7–22.9	31.2	21.0	
Chemotherapy					<0.001
No (*n* = 179)	16.0	11.3–20.6	29.1	20.3	
Yes (*n* = 655)	24.5	22.0–27.0	38.1	28.7	

*CI* confidence interval, *LN* ratios, ratios of metastatic to examined lymph nodes, *MC* mucinous carcinoma, *PDC* poorly differentiated carcinoma, *SRCC* signet ring cell carcinoma

**Table 4:** Multivariate analysis of prognostic factors for stage III undifferentiated gastric adenocarcinoma

Factors	Hazard ratio (HR)	95 % CI for HR		*P* value
		Lower	Upper	
Type of gastrectomy				
Total/subtotal	1.345	1.130	1.602	0.001
Tumor size (cm)				
>5/≤5	1.251	1.049	1.492	0.013
LN ratio				
>0.34/≤0.34	2.262	1.892	2.704	<0.0001
Resection margins				
R1/R0	1.538	1.238	1.911	<0.001
Histology type				
PDC/MC	1.594	1.012	2.510	0.044
SRCC/MC	1.518	0.949	2.429	0.081
Lymphatic invasion				
Yes/no	1.045	0.813	1.343	0.732
Vascular invasion				
Yes/no	0.980	0.800	1.201	0.845
Perineural invasion				
Yes/no	1.335	1.095	1.627	0.004
Chemotherapy				
No/yes	1.381	1.125	1.694	0.002

*CI* confidence interval, *LN* ratios, ratios of metastatic to examined lymph nodes, *MC* mucinous carcinoma, *PDC* poorly differentiated carcinoma, *SRCC* signet ring cell carcinoma

### Cumulative survival rates

**Fig. 1:**
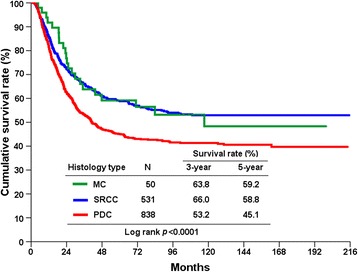
Cumulative overall survival (OS) rates in stage I–III gastric cancer according to histology type

**Fig. 2:**
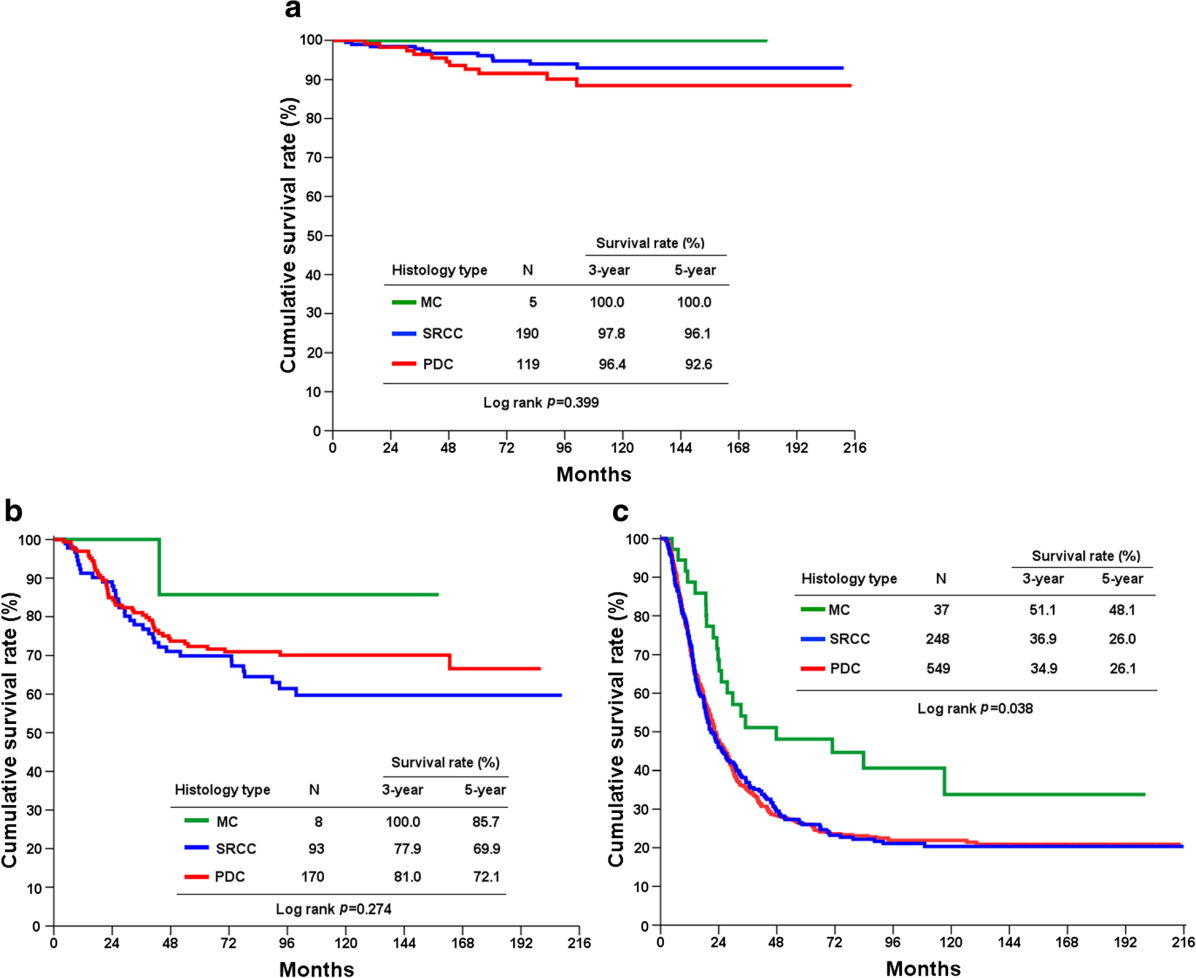
Cumulative overall survival (OS) rates in stage I (**a**), II (**b**), and III (**c**) gastric cancer according to histology type

The 5-year overall survival (OS) rates of stage I–III undifferentiated GC patients undergoing potentially curative resection were 58.8, 59.2, and 45.1 % in SRCC, MC, and PDC, respectively (Fig. 1; *P* < 0.0001). Similar OS rates were found for stage I (Fig. 2a; *P* = 0.399) and stage II (Fig. 2b; *P* < 0.274) patients in the three groups. Compared to stage III SRCC and PDC patients, MC patients had markedly favorable OS rates (Fig. 2c; *P* = 0.038).

## Discussion

In accordance with previous reports [6, 10], MC of the stomach is a rare histologic type of GC, comprising 4.4 % of the cases of undifferentiated stage I–III GC in our study. In potentially curative resected GC (stages I–III), MC patients were older than SRCC or PDC patients and the tumors in MC patients were larger than those in SRCC or PDC patients. Patients with MC were more often diagnosed as stage III compared with the other undifferentiated subtypes. In stage III GC, the age and size of MC were significantly older and larger than SRCC or PDC, respectively; lower proportions of perineural invasion were identified in MC, and female predominance was noted in SRCC in comparison with the other two subtypes. The cumulative OS rates of stage I–III GC patients with MC were significantly longer compared to those with PDC, but not SRCC. Stage III GC patients with MC had a better prognosis than those with SRCC or PDC; the difference in survival was not evident in stage I or II patients.

Although studies have reported the distinct clinicopathological features of MC compared with non-MC, the results remain inconsistent. Kawamura et al. indicated that the patients with MC were younger than the non-MC patients [11]. Yin et al. suggested that age had no relationship with MC and non-MC and MC was associated with a larger tumor size than non-MC [6]. Other studies found that there was no difference in the tumor size between patients with MC and non-MC [7, 12]. Kunisaki et al. also noted that compared with non-MC, MC had deeper invasion and more lymph node metastasis [7]. In addition, more advanced stages were identified in MC at the time of diagnosis compared with non-MC [6, 7, 10, 11, 13]. In this study, we only included stage I–III resected undifferentiated GC and compared the clinicopathological characteristics of MC with SRCC or PDCC. Significant differences were noted in age; sex; size; depth of tumor invasion; nodal involvement; disease stage; and presence of lymphatic invasion, vascular invasion, and perineural invasion among patients with MC, SRCC, and PDC. In the subgroup analysis of stage III disease, our results showed that older patients, larger tumor size, and higher percentages of perineural invasion were found in those with MC histology compared with SRCC or PDC subtypes; SRCC and PDC had female and male predominance, respectively.

The prognosis of patients with undifferentiated GC compared with other histology is still controversial. Our previous studies have shown that early GC patients with SRCC had more favorable survival than those with non-SRCC; however, advanced SRCC resulted in significantly worse survival than non-SRCC [14]. Interestingly, Kwon et al. found that survival in early GC patients exhibited no difference between histological types; advanced GC patients with SRCC had a worse prognosis than those with other histological types [15]. Furthermore, Shim et al. reported that SRCC is not an independent predictor of poor prognosis after curative resection for GC [16]. Park et al. indicated that histological type was not statistically associated with survival in stage I, II, or III patients in stage-stratified analysis [5]. Similarly, the prognosis of MC did not differ from non-MC for each stage [6]. In contrast, Fan et al. reported that stage I and II MC patients had a worse 5-year OS than those with SRCC (*P* = 0.012); a difference in 5-year OS was not evident between stage III SRCC and MC groups [17]. In the present study, we examined the outcomes of undifferentiated GC patients undergoing curative intent surgery. Our results, based on stage-stratified analysis, indicated that stage III GC patients with MC had a better prognosis than those with PDC or SRCC (Fig. 2c; *P* = 0.038); the difference in OS was not evident in stage I or II patients (Fig. 2a, b). Importantly, MC is an independent prognostic factor in multivariate analysis in stage III disease (PDC vs. MC; HR = 1.594; *P* = 0.044; Table 4), which is different from previous reports [9].

Although stage III patients with MC had an older age and larger tumor size than SRCC or PDC patients, less perineural invasion was identified in MC, which might in part explain the favorable outcome of MC over other undifferentiated subtypes in the current study. In this regard, Deng et al., using meta-analysis methodology, indicated that perineural invasion is an independent poor prognostic factor in radically resected GC [18]. In line with their findings, our results also showed that perineural invasion is an independent predictor for worse survival in stage III undifferentiated GC in multivariate analysis.

Our results showed that MC had higher percentages of stage III disease compared with SRCC or PDC. In this regard, previous studies suggested that MC is believed to arise initially as a typical adenocarcinoma that becomes mucinous as the tumor progresses [12]. Furthermore, the intra-luminal secretion of mucin decreases and the deposit of mucin increases, resulting in intra-luminal accumulation when the tumor invades the gastric wall [19]. Other researchers have noted that MC over-expressed mucin 2 and oligomeric mucus/gel-forming proteins compared with non-MC [10]. In addition, Choi et al. showed that MC presented statistically lower levels of β-catenin and a more advanced stage than non-MC [13]. Nonetheless, more studies are needed to clarify the biological behavior and histogenesis of MC.

## Conclusions

MC is a rare type of GC. Our results indicated that stage I–III GC with a mucinous subtype presented with different clinicopathological features (older age and larger tumor size) and a different prognosis than SRCC and PDC subtypes. Patients with MC are more frequently diagnosed with stage III disease compared with other undifferentiated subtypes. There was no difference in survival for stages I or II among MC, SRCC, and PDC. Stage III gastric MC had significantly better survival than SRCC or PDC.

## Abbreviations

GC: gastric adenocarcinoma
MC: mucinous carcinoma
OS: overall survival
PDC: poorly differentiated carcinoma
SRCC: signet ring cell carcinoma
